# A trustworthy mechanochemical route to isocyanides

**DOI:** 10.3762/bjoc.18.73

**Published:** 2022-06-22

**Authors:** Francesco Basoccu, Federico Cuccu, Federico Casti, Rita Mocci, Claudia Fattuoni, Andrea Porcheddu

**Affiliations:** 1 Dipartimento di Scienze Chimiche e Geologiche, Università degli Studi di Cagliari, Cittadella Universitaria, Monserrato, 09042 Cagliari, Italy

**Keywords:** green chemistry, isocyanide, isonitriles, mechanochemistry

## Abstract

Isocyanides are hardly produced, dramatically sensitive to purification processes, and complex to handle as synthetic tools. Notwithstanding, they represent one of the most refined and valuable compounds for accessing sophisticated and elegant synthetic routes. A unique interest has always been addressed to their production, though their synthetic pathways usually involve employing strong conditions and toxic reagents. The current paper intends to provide a conceptually innovative synthetic protocol for mechanochemical isocyanide preparation, simultaneously lowering the related reagents' toxicity and improving their purification in a straightforward procedure.

## Introduction

**Scheme 1:**
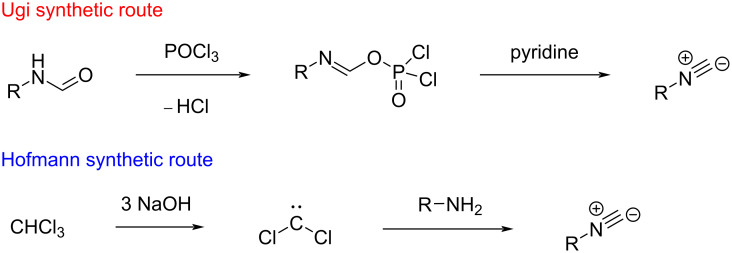
Historic synthetic approaches.

Isocyanides were first discovered more than a century ago by Lieke in Göttingen after having handled allyl iodide and potassium cyanide to synthesize crotonic acid. This attempt, instead, brought to the accidental synthesis of an isocyanide which was recognizable by its revolting smell, as reported by Lieke [1]. A deepening of the topic arrived, though, only in the following century, when a natural and potential pharmaceutical compound was discovered, namely xanthocillin [2]. Finally, after two decades, the first synthetic approaches were reported by Ugi and Hofmann (Scheme 1) [3–4], who described their characteristic odour as “horrible” and “extremely distressing”. With such a breakthrough, isonitriles gained wide popularity in organic synthesis due to their extreme versatility [5–7]. Especially, they are often used in heterocycles formation [8–9], multicomponent strategies [10–11], polymers production [12–13], and metal complexation [14–15].

**Figure 1:**
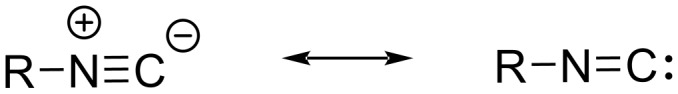
Resonance forms of isocyanides.

The molecular structure is composed of an N–C planar triple bond where the nitrogen atom assumes a positive charge due to the fourth bond with a carbon chain, which could be aliphatic or aromatic. In contrast, the carbon of the functional group bears a negative localized charge [16]. Apparently unstable and unreasonable, this is the most likely and plausible resonance structure because the other hypothesised form, where the carbenic counterpart does not respect the octet rule, is less favoured (Figure 1).

**Scheme 2:**
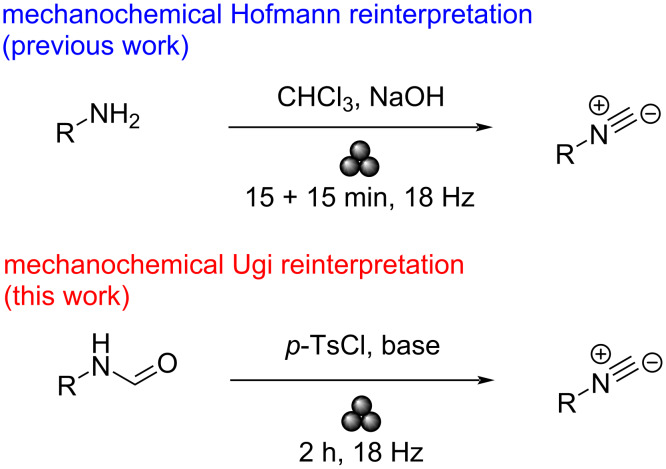
Comparison between the previous mechanochemical synthetic pathway [24] and the new adapted one in this work.

The synthetic approaches to this core are multiple, but numerous drawbacks severely limit their output. Common pathways generally involve formamides; one of the most known is Ugi’s method [17] which is based on the dehydration of a primary formamide with POCl_3_ in the presence of a base. Apart from phosphorus-mediated compounds, phosgene or diphosgene [18] work well, despite being still affected by the same limitations of POCl_3_. Therefore, organic chemists decided to move to other safer shores, so different dehydrating agents were also proposed. Tosyl chloride [19–20] and TCT (1,3,5-trichlorotriazine) [21] proved to be valuable alternatives to the aforementioned phosphorous compounds due to their powerful dehydrating ability. Lastly, Burgess reagent has been reported as a mild and selective dehydrating compound for formamides [22–23]. As we previously mentioned, the Hofmann isocyanide synthesis is a historical approach which our group revised mechanically [24]. Following this line, an eco-friendlier Ugi’s isocyanide synthesis will be depicted in this article (Scheme 2).

## Results and Discussion

At the beginning of this project, we envisioned the feasibility of producing isocyanides from primary formamides by using anhydrides as dehydrating agents. Therefore, we focused our attention on acetic, trifluoroacetic, and isatoic anhydrides to achieve this. First attempts were made on acetic and trifluoroacetic anhydrides. The best results were obtained when a stoichiometric ratio between the anhydride and the reference compound, *N*-benzylformamide (**1f**), was milled with 2 equivalents of triethylamine or *N*-methylimidazole as bases. Despite slightly performing as a method, the reaction mixture was “too liquid” for running a mechanochemical reaction. This is why a solid anhydride, like the isatoic one, captured our interest, but unfortunately, the results were unsatisfying.

A second idea relied on traditional coupling reagents, especially carbodiimides (DIC and DCC) [25] and CDI [26–29]. The former should generate a urea derivative whilst the latter should produce CO_2_ and imidazole as byproducts. In both cases, the reaction driving force is the production of thermodynamically stable products. However, they did not bring any advantage compared to acetic anhydride. Considering that CDI is activated in an acid environment, adding a catalytic amount of NaHSO_4_ was necessary. Since isocyanides are sensitive to acids, this approach was ruled out a priori. Aware of these issues, we were discouraged from employing *p*-tosylimidazole as well, since it requires acid activation to be effective as a dehydrating agent.

Accordingly, we moved our interest to DIC and DCC compounds, used in stoichiometric quantity and in the presence of 1 equivalent of NEt_3_. Unfortunately, their use formed the desired product only in traces, so we finally decided to converge our efforts on the use of tosyl chloride as previously reported [19,30]. We found in it the most suitable agent for synthesising isocyanides when combined with a basic milieu. Among the immense variety of compounds that can be employed as bases, only a few are reported to catalyse such a process: pyridine [31] and triethylamine [32] are the most representative ones. Their reactivity can be attributed to their probable reaction mechanism where the nitrogen atom not only promotes the enolate derivative formation as already described in literature [33–34] but it may also generate a positively charged intermediate at the transition state [35], enhancing the nucleophilic substitution. Obviously, pyridine handling is associated with many risks, mainly concerning human health [36]. Consequently, our idea was to substitute pyridine with *N*-methylimidazole because their basicity and physical state are analogous. When the reactions between the formamide and different equivalents (from 1 to 6) of *N*-methylimidazole were carried out, the outcomes were not as good as those already documented with pyridine in the literature. Possible explanations for this phenomenon are either a different electronic distribution between the two heterocycles or the absence of intermolecular interactions caused by the solvent.

The association of triethylamine, *p*-tosyl chloride, and **1f** gave the best results, so, following this trend, we started the optimisation process. First attempts involved a 1:1:2 ratio between formamide **1f**, *p*-tosyl chloride, and triethylamine which resulted in an approximately 40% yield of isocyanide **2f** (GC–MS analysis). Unexpectedly, the addition of Lewis acids, namely LiCl and BF_3_**·**Et_2_O, did not improve the enolate generation. The next step was searching the optimal conditions for a better conversion of **1f** in **2f**. These were found in the 1:2:7 ratios of the three components with the addition of 400 mg of NaCl as a grinding auxiliary (0.5 h, 36 Hz, Table S1 in Supporting Information File 1). So, we then looked for a solid base to use in place of triethylamine, avoiding the use of further additives. Unluckily, neither solid inorganic bases such Na, K, and Cs carbonates [37], Mg and Ba oxides, nor organic bases like potassium *tert*-butoxide and imidazole proved to be as effective as triethylamine. Much to our surprise, we observed that the association between only 1 equivalent of triethylamine and 6 equivalents of sodium carbonate brought a good conversion rate of **1f** in **2f** at a frequency of 36 Hz after 1h (70%, GC–MS analysis). Reducing the amount of sodium carbonate did not provide any advantage. Interestingly, using anhydrous sodium carbonate instead of its hydrated form improved reaction yields, likely due to the hydrolysis of tosyl chloride. With these data in hand, we then opted for refining other mechanochemical parameters such as frequency and reaction time. We attempted to emulate milder operating conditions as previously established in our paper [24] because of their high degradation rate [38] and instability [39]. Our idea found a match in the experimental data, confirming the total conversion of **1f** to **2f** after 1 h at 18 Hz.

**Scheme 3:**
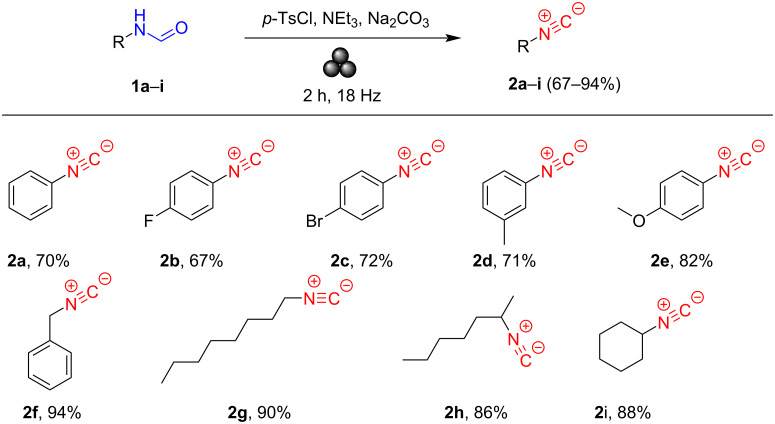
The scope of our isocyanide synthesis using aliphatic and aromatic primary formamides. Reaction conditions: formamide **1a**–**i** (1.0 mmol), *p*-TsCl (1.5 mmol), triethylamine (1.0 mmol), dry Na_2_CO_3_ (6.0 mmol), 1 h, zirconia jar (15 mL), 2 balls (Ø = 8 mm), 18 Hz.

With this comprehensive insight, we applied the described method to both aliphatic and aromatic substrates **1a**–**i** (Scheme 3). The conversion was almost complete for the aliphatic compounds **1f**–**i**, with yields from very high to excellent. On the other side, for the aromatic starting compounds **1a**–**e**, the reaction did not exceed the maximum of 82% yield obtained for **2e**. It should be emphasized that the preparation of aromatic isocyanides has always been a challenging process from a synthetic point of view. Such a different fashion can be ascribed to the diverse electronic distribution between aliphatic and aromatic formamides. Concerning aromatic amides, the presence of electron-withdrawing (EWG) or electron-donating groups (EDG) further affect the tautomeric equilibrium, promoting or weakening the reactivity of the substrates. In this case, the yields are high for EDGs (**2d**,**e**), while from good to high for the EWGs (**2b**,**c**). To confirm what has been previously stated, isonitrile **2a** was recovered in lower yields than compound **2e** (Scheme 3).

At the end of the reaction, adding 0.5 equivalents of water for a 15 minute grinding step was necessary to hydrolyse the *p*-tosyl chloride excess. Our approach for the removal of the unreacted *p*-tosyl chloride was demonstrated to be the most feasible and eco-friendly compared with the reported techniques, which imply the use of cellulose with a large excess of pyridine [40] and the need for flash chromatography [41].

**Figure 2:**
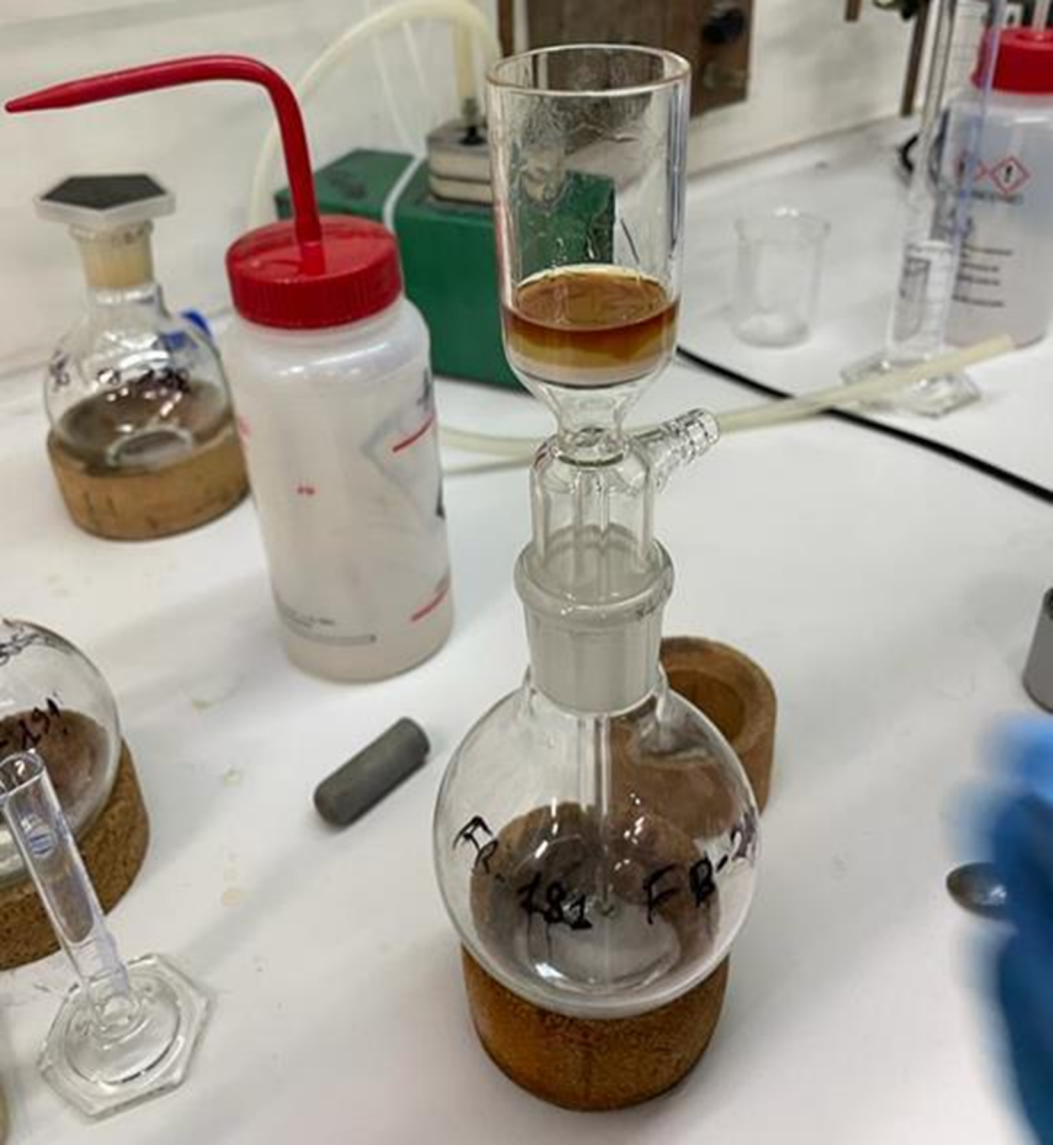
The purification process of a brownish isocyanide on a short silica pad.

Afterwards, the mixture was recovered as a solid in a beaker, shredded in *n*-heptane, and filtered on paper. The organic solution only contained the desired product and various quantities of starting material, depending on the formamide employed. A short silica pad was then used to increase the isocyanide purity (Figure 2), even though, in some cases, a small amount of sulphonyl derivatives or solvent residues can be spotted. These little impurities are due to the isocyanide instability and difficulty in being held.

**Scheme 4:**
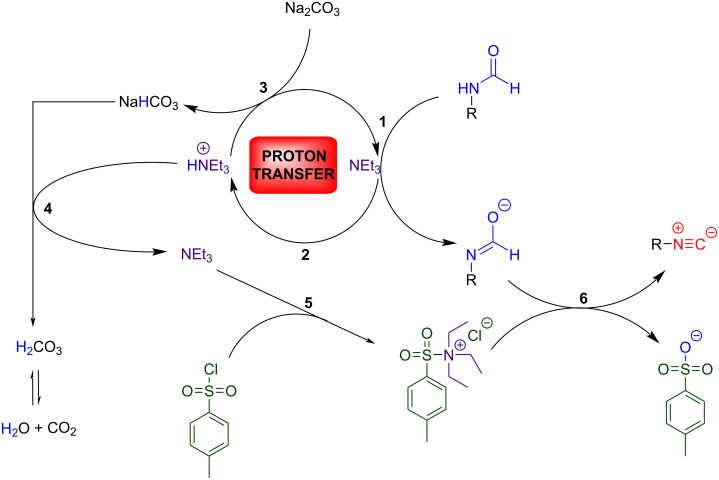
Suggested proton transfer mechanism.

In light of what was said above, it makes sense to hypothesise a possible reaction mechanism (Scheme 4). The single equivalent of triethylamine should be able to activate the tautomerism of the formamide through an acid–base reaction [33,42–45]. The triethylammonium salt produced can be restored as triethylamine through the action of Na_2_CO_3_. This proton transfer allows the formation of NaHCO_3,_ which should still be sufficiently basic for deprotonating again the regenerated ammonium species, releasing H_2_O in the process.

## Conclusion

Even though there is a tremendous interest in the synthesis of isocyanides, only few procedures have been developed since their discovery. Since so many troublesome downsides characterise them, any possible alternative synthetic route has been unfairly put aside. Nonetheless, we demonstrated the feasibility of their synthesis through a greener procedure employing cheap reagents such as sodium carbonate [46] and waste materials deriving from industry such as TsCl (mechanochemical approach: EcoScale → 81; E-Factor → 9.5; in-solution approach: EcoScale → 55; E-Factor → 131.1; see Supporting Information File 1 for more details) [47–48]. Not only did we plummet the reaction expenditures, but we also undoubtedly proved that our method could be entirely exerted in the solid phase through mechanochemical activation. In conclusion, we hope that our synthetic strategy could be considered a significant step toward a less impacting and more extensive research on isocyanide chemistry.

## Supporting Information

File 1 Experimental.
