# Supplementary material for: A trustworthy mechanochemical route to isocyanides
Source: Beilstein J Org Chem. 2022 Jun 22;18:732–7. doi: 10.3762/bjoc.18.73 (PMC9235834; doi:10.3762/bjoc.18.73)
Supplement: File 1 — Experimental. [file Beilstein_J_Org_Chem-18-732-s001.pdf]

BEILSTEIN JOURNAL OF ORGANIC CHEMISTRY

Supporting Information

for

A trustworthy mechanochemical route to isocyanides

Francesco Basoccu, Federico Cuccu, Federico Casti, Rita Mocci, Claudia Fattuoni and Andrea Porcheddu

Beilstein J. Org. Chem. **2022,** *18,* 732–737. doi:10.3762/bjoc.18.73

Experimental

License and Terms: This is a supporting information file under the terms of the Creative Commons Attribution License (https://creativecommons.org/licenses/by/4.0). Please note that the reuse, redistribution and reproduction in particular requires that the author(s) and source are credited and that individual graphics may be subject to special legal provisions.
The license is subject to the *Beilstein Journal of Organic Chemistry* terms and conditions: (https://www.beilstein-journals.org/bjoc/terms)

Table of contents

1. General information S2
2. Mechanochemical synthesis of **2a**–**i** S3
3. Procedure for the optimization of the reaction S7
4. Green chemistry metrics calculations S8
5. References S12
6. NMR spectra S13

1. General information

Commercially available reagents were purchased from Acros, Aldrich, Strem Chemicals, Alfa-Aesar, TCI Europe and used as received. All reactions were monitored by thin-layer chromatography (TLC) performed on glass-backed silica gel 60 F254, 0.2 mm plates (Merck), and compounds were visualized under UV light (254 nm) or using cerium ammonium molybdate solution with subsequent heating. The eluents were technical grade. Mechanochemical reactions were carried out using a FormTech FTS-1000 Shaker Mill® apparatus. The reagents were milled using a zirconia SmartSnap™ grinding jar (15 mL) equipped with balls (Ø = 8 mm) of the same material. Precisely, the zirconium oxide of the vessels and balls used for all reactions accomplished in the mixer mill is yttrium oxide stabilized (ZrO2-Y). These parameters were applied if not stated otherwise. ^{1}H and ^{13}C liquid NMR spectra were recorded on a Varian 500 MHz and Bruker Avance III HD 600 MHz NMR spectrometer at 298 K and were calibrated using trimethylsilane (TMS). Proton chemical shifts are expressed in parts per million (ppm, δ scale) and are referred to the residual hydrogen in the solvent ($CHCl_3$, 7.27 ppm or DMSO 2.54 ppm). Data are represented as follows: chemical shift, multiplicity (s = singlet, d = doublet, t = triplet, q = quartet, m = multiplet and/or multiple resonances, br s = broad singlet, and combination of thereof), coupling constant (J) in hertz (Hz) and integration. Carbon chemical shifts are expressed in parts per million (ppm, δ scale) and are referenced to the carbon resonances of the NMR solvent ($CDCl_3$, δ 77.0 ppm or δ DMSO-d_6 δ 39.5 ppm). Deuterated NMR solvents were obtained from Aldrich. Samples were analyzed using an Agilent 5977B MS interfaced to the GC 7890B equipped with a DB-5ms column (J & W), injector temperature at 230 °C, detector temperature at 280 °C, helium carrier gas flow rate of 1 ml/min. The GC oven temperature program was 60 °C initial temperature with 4 min hold time and ramping at 15 °C/min to a final temperature of 270 °C with 7 min hold time. One µL of each sample was injected in split (1:20) mode. After a solvent delay of 3 min mass spectra were acquired in full scan mode using 2.28 scans/s with a mass range of 50–500 Amu. Retention times of different compounds were determined by injecting pure compounds under identical conditions. All the experiments were carried out in duplicate to ensure reproducibility of the experimental data. Yields refer to pure isolated materials.

2. Mechanochemical synthesis of isocyanides 2a–i

General procedure for isocyanide synthesis

A 15 mL ZrO_2 jar equipped with two ZrO_2 milling balls (8.0 mm in diameter) was filled with formamide **1a–i** (1.0 mmol), *p*-tosyl chloride (1.5 mmol), triethylamine (1.0 mmol), and Na_2CO_3 (6.0 mmol). The vessel was then closed and the mechanochemical reaction was conducted for 60 min at a frequency of 18 Hz. At the end of the reaction, additional 15 min grinding with H_2O (0.5 equiv) was performed. After this step, the crude was recovered as a solid in a beaker and dissolved in heptane (10 mL). A short silica pad (1.0 g) was required for a further purification. Lastly, the organic layer was dried over Na_2SO_4, and the solvent was removed under reduced pressure to afford the pure isocyanide compound.

General procedure for isocyanide gram scale synthesis

A 15 mL ZrO_2 jar equipped with three ZrO_2 milling balls (10 mm in diameter) was filled with formamide **1a**–**i** (5.0 mmol), *p*-tosyl chloride (7.5 mmol), triethylamine (5.0 mmol), and Na_2CO_3 (30 mmol). The vessel was then closed and the mechanochemical reaction was conducted for 60 min at a frequency of 18 Hz. At the end of the reaction, additional 15 min grinding with H_2O (0.5 equiv) was performed. After this step, the crude was recovered as a solid in a beaker and dissolved in heptane (50 mL). A short silica pad (5.0 g) was required for a further purification. Lastly, the organic layer was dried over Na_2SO_4, and the solvent was removed under reduced pressure to afford the pure isocyanide compound.

Isocyanobenzene (**2a**)

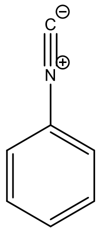

The title compound was synthesized according to the general procedure stated above. **1a** (121.1 mg, 1.0 mmol), p-tosyl chloride (285.9 mg, 1.5 mmol), triethylamine (139 µl, 1.0 mmol), and Na_2CO_3 (635.9 mg, 6.0 mmol) were used, to afford **2a** after a short silica pad (SiO_2/*n*-heptane 100% v/v) as a yellowish oil (72.2 mg, 0.7 mmol, 70%).

R_f = 0.60 (*n*-heptane:EtOAc 9:1 v/v)

^{1}H NMR (600 MHz, $CDCl_3$): δ = 7.40-7.36 (m, 5H)

^{13}C NMR (151 MHz, $CDCl_3$): δ = 164.1, 129.6, 129.5, 126.6.

The spectroscopic data closely match the ones previously reported in the literature.[1]

1-Fluoro-4-isocyanobenzene (**2b**)

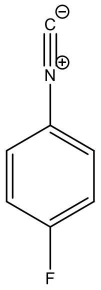

The title compound was synthesized according to the general procedure stated above. **1b** (139.1 mg, 1 mmol), p-tosyl chloride (285.9 mg, 1.5 mmol), triethylamine (139 µl, 1 mmol), and Na_2CO_3 (635.9 mg, 6 mmol) were used, to afford **2b** after a short silica pad (SiO_2/n-heptane 100% v/v) as a brownish solid (81.1 mg, 0.67 mmol, 67%).

R_f = 0.64 (*n*-heptane:EtOAc 9:1 v/v)

^{1}H NMR (600 MHz, $CDCl_3$): δ = 7.38-7.36 (d, *J* = 8.5 Hz, 2H), 7.32-7.31 (d, *J* = 8.4 Hz, 2H).

^{13}C NMR (151 MHz, $CDCl_3$): δ = 165.8, 135.5, 129.9, 127.8, 125.3, 125.2, 125.1.

The spectroscopic data closely match the ones previously reported in the literature.[2]

1-Bromo-4-isocyanobenzene (**2c**)

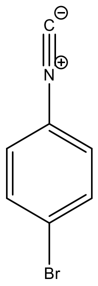

The title compound was synthesized according to the general procedure stated above. **1c** (200.0 mg, 1.0 mmol), *p*-tosyl chloride (285.9 mg, 1.5 mmol), triethylamine (139 µl, 1.0 mmol), and Na_2CO_3 (635.9 mg, 6.0 mmol) were used, to afford **2c** after a short silica pad (SiO_2/n-heptane 100% v/v) as a yellowish solid (131.0 mg, 0.72 mmol, 72%).

R_f = 0.61 (*n*-heptane:EtOAc 9:1 v/v)

^{1}H NMR (600 MHz, $CDCl_3$): δ = 7.55 – 7.53 (m, 2H), 7.26-7.25 (d, *J* = 2.1 Hz, 2H).

^{13}C NMR (151 MHz, $CDCl_3$): δ = 132.9, 128.0, 123.6.

The spectroscopic data closely match the ones previously reported in the literature.[3]

1-Isocyano-3-methylbenzene (**2d**)

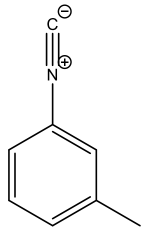

The title compound was synthesized according to the general procedure stated above. **1d** (135.2 mg, 1 mmol), p-tosyl chloride (285.9 mg, 1.5 mmol), triethylamine (139 mg, 1 mmol), and Na_2CO_3 (635.9 mg, 6 mmol) were used, to afford **2d** after a short silica pad (SiO2/n-heptane 100% v/v) as a brown oil (83.2 mg, 0.71 mmol, 71%).

R$_f$ = 0.57 (*n*-heptane:EtOAc 9:1 v/v)

^{1}H NMR (600 MHz, CDCl$_3$): δ = 7.27 – 7.25 (m, 1H), 7.21 – 7.17 (m, 3H), 2.36 (s, 3H).

^{13}C NMR (151 MHz, CDCl$_3$): δ = 163.7, 139.8, 130.3, 129.3, 127.0, 123.6, 21.2.

1-isocyano-4-methoxybenzene **2e**

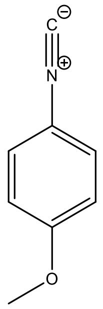

The title compound was synthesized according to the general procedure stated above. **1e** (151.2 mg, 1.0 mmol), *p*-tosyl chloride (285.9 mg, 1.5 mmol), triethylamine (139 µl, 1.0 mmol), and Na_2CO_3 (635.9 mg, 6.0 mmol) were used, to afford **2e** after a short silica pad (SiO2/n-heptane 100% v/v) as a yellow solid (109.2 mg, 0.82 mmol, 82%).

R$_f$ = 0.45 (*n*-exane:EtOAc 9:1 v/v)

^{1}H NMR (600 MHz, CDCl3): δ = 7.31 (d, 2H), 6.93 – 6.83 (m, 2H), 3.82 (s, 3H).

^{13}C NMR (151 MHz, CDCl3): δ = 160.0, 127.9, 114.7, 55.7.

The spectroscopic data closely match the ones previously reported in the literature.[1]

Benzyl isocyanide (**2f**)

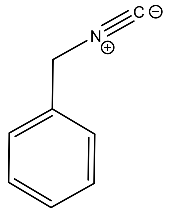

The title compound was synthesized according to the general procedure stated above. **1f** (135.2 mg, 1.0 mmol), *p*-tosyl chloride (285.9 mg, 1.5 mmol), triethylamine (139 µl, 1.0 mmol), and Na_2CO_3 (635.9 mg, 6.0 mmol) were used, to afford **2f** after a short silica pad (SiO2/n-heptane 100% v/v) as a brown oil (110.2 mg, 0.94 mmol, 94%).

R$_f$ = 0.55 (*n*-heptane:EtOAc 9:1 v/v)

^{1}H NMR (600 MHz, CDCl$_3$): δ = 7.44 – 7.38 (m, 2H), 7.38 – 7.32 (m, 3H), 4.65 (t, *J* = 2.3 Hz, 2H).

^{13}C NMR (151 MHz, CDCl$_3$): δ = 157.8, 132.35, 129.00, 128.44, 126.62, 45.6.

The spectroscopic data closely match the ones previously reported in the literature.[1]

1-Isocyanooctane (**2g**)

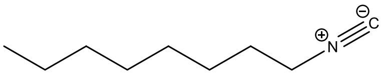

The title compound was synthesized according to the general procedure stated above. **1g** (157.3 mg, 1.0 mmol), *p*-tosyl chloride (285.9 mg, 1.5 mmol), triethylamine (139 µl, 1.0 mmol), and Na_2CO_3 (635.9 mg, 6.0 mmol) were used, to afford **2g** after a short silica pad (SiO2/n-heptane 100% v/v) as a colourless oil (125.3 mg, 0.9 mmol, 90%)

$\mathbf{R_f}$ = 0.53 (*n*-heptane:EtOAc 9:1 v/v)

^{1}H NMR (600 MHz, $CDCl_3$): δ = 3.37 (ddt, 2H), 1.67 (dddd, 2H), 1.43 (td, 2H), 1.35 – 1.21 (m, 8H), 0.89 (t, 3H).

^{13}C NMR (151 MHz, $CDCl_3$): δ = 155.7, 41.8, 41.7, 41.6, 31.8, 29.3, 29.2, 28.8, 26.5, 22.7, 14.2.

The spectroscopic data closely match the ones previously reported in the literature.[1]

2-Isocyanoheptane (**2h**)

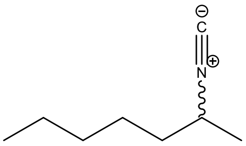

The title compound was synthesized according to the general procedure stated above. **1h** (143.1 mg, 1.0 mmol), *p*-tosyl chloride (285.9 mg, 1.5 mmol), triethylamine (139 µl, 1.0 mmol), and Na_2CO_3 (635.9 mg, 6.0 mmol) were used, to afford **2h** after a short silica pad (SiO2/n-heptane 100% v/v) as a yellow oil (107,6 mg, 0.86 mmol, 86%).

$\mathbf{R_f}$ = 0.50 (*n*-heptane:EtOAc 9:1 v/v)

^{1}H NMR (600 MHz, $CDCl_3$): δ = 3.62-3.58 (m, 1H), 1.70-1.69 (m, 2H), 1.36-1.34 (m, 6H), 0.91-0.88 (m, 6H).

^{13}C NMR (151 MHz, $CDCl_3$): δ = 154.2, 50.4, 36.9, 31.3, 25.5, 22.6, 21.8, 14.1.

Isocyanocyclohexane (**2i**)

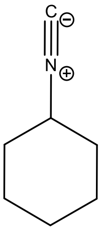

The title compound was synthesized according to the general procedure stated above. **1i** (127.2 mg, 1.0 mmol), *p*-tosyl chloride (285.9 mg, 1.5 mmol), triethylamine (139 µl, 1.0 mmol), and Na_2CO_3 (635.9 mg, 6.0 mmol) were used, to afford **2i** after a short silica pad (SiO2/n-heptane 100% v/v) as a yellowish oil (96.1 mg, 0.88 mmol, 88%).

$\mathbf{R_f}$ = 0.60 (*n*-heptane:EtOAc 9:1 v/v)

^{1}H NMR (600 MHz, $CDCl_3$): δ = 3.61-3.58 (tt, *J* = 8.2, 3.4 Hz, 1H), 1.90 – 1.86 (m, 2H), 1.77 – 1.64 (m, 4H), 1.49-1.34 (m, 4H).

^{13}C NMR (151 MHz, $CDCl_3$): δ = 154.1, 52.0, 51.9, 51.8, 32.8, 25.1, 22.9.

The spectroscopic data closely match the ones previously reported in the literature.[1]

3. Procedure for the optimization of the reaction (Table S1)

A 15 mL ZrO_2 jar equipped with two ZrO_2-Y milling ball (8.0 mm diameter) was filled with formamide **1f** (1.0 mmol), dehydrating combo reagents. The vessel was then closed and the mechanochemical reaction was conducted at different times in a different range of frequency (from 18 to 36 Hz). At the end of the reaction, the crude was recovered as a solid in a beaker, dissolved in hexane (15 mL). A silica pad (2.0 g) was required for purification. Lastly, the organic layer was dried over Na_2SO_4, and the solvent was removed under reduced pressure to afford the pure isocyanide compound.

Table S1. Optimization of the reaction conditions for isocyanide **2f** synthesis in the presence of different dehydrating agents.

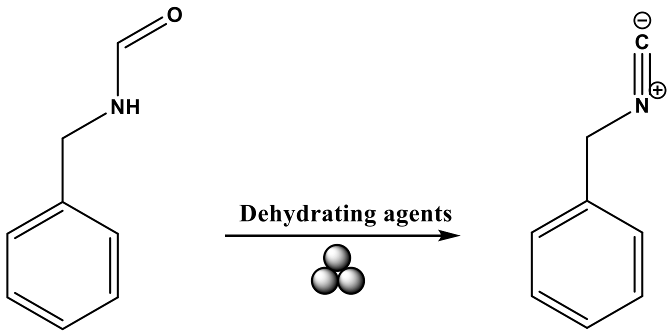

Entry	Dehydrating agents	Additive	Yield (%)[g]
1	Ac_2O	Triethylamine[a]	60%
2	TFAA	Triethylamine[a]	67%
3	Isatoic Anhydride	Triethylamine[a]	< 1%
4	CDI	$NaHSO_4$	traces
5	DIC	Triethylamine[a]	18%
6	p-Ts imidazole	$NaHSO_4$	traces
**7**	p-Ts imidazole	$NaHSO_4$[a]	traces
8	**p-TsCl**	**Triethylamine**[d,e]	**quant.**
9	p-TsCl	N-methyl imidazole	43%
10	p-TsCl	N-methyl imidazole[b,c,e]	75%
11	p-TsCl	Imidazole[b,c]	20%
12	p-TsCl	Na_2CO_3 anydrous + triethylamine	70%
13	**p-TsCl**	**Na_2CO_3 anydrous + Triethylamine**[e,f]	**94%**
14	p-TsCl	Na_2CO_3 anhydrous	2%
15	p-TsCl	Na_2CO_3	< 1%
16	p-TsCl	K_2CO_3	traces
17	p-TsCl	Cs_2CO_3	traces
18	p-TsCl	MgO	traces
19	p-TsCl	BaO	traces
20	p-TsCl	LiCl	traces
21	p-TsCl	$BF_3{\cdot}Et_2O$	Traces

Reaction conditions: formamide **1f** (1 mmol), dehydrating agents (1 mmol), additive (10 mol%), 60 min, 36 Hz. [a] Additive (1 mmol). [b] Additive (2 mmol). [c]120 min reaction. [d]additive (7 mmol), NaCl (400 mg), 0.5 h, 36 Hz. [e] Frequency of 18 Hz. [f] Na_2CO_3 (6 mmol), triethylamine (1 mmol). [g]GC-MS calculated yields.

4. Green chemistry metrics calculations

Calculation of the Green Chemistry metrics for the mechanochemical preparation of **1f**

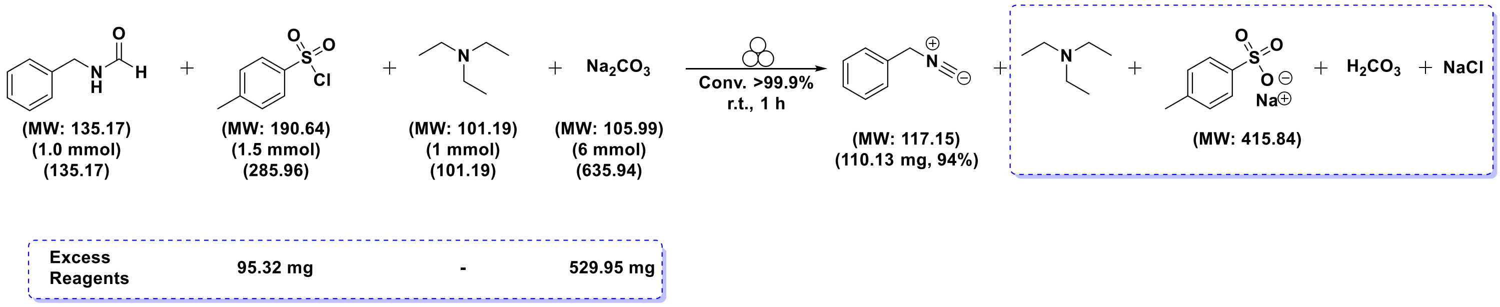

Scheme S1. Mechanochemical preparation of Isocyanide **1f**.

Calculation of Green Chemistry Metrics

$$\textbf{A}\text{tom }\textbf{E}\text{conomy} = \frac{\text{Mass of desired useful product}}{\text{Total Mass of all reactants}} \times 100 = \frac{117{,}15}{532{,}99} \times 100 = 22\%$$

$$\textbf{E}\text{nvironmental }\textbf{F}\text{actor} = \frac{\text{Mass of total waste}}{\text{Mass of desired product}} = \frac{194{,}18 + 95{,}32 + 58.44 + 101{,}19 + 529{,}95 + 62.03}{110{,}13} = 9{,}5$$

$$\textbf{R}\text{eaction }\textbf{M}\text{ass }\textbf{E}\text{fficiency} = \frac{\text{actual mass of desired product}}{\text{mass of reactants}} \times 100 = \frac{110{,}1}{135{,}2 + 286{,}0 + 101{,}2 + 635{,}9} \times 100 = 9{,}5\%$$

The eco-scale score for the mechanochemical preparation of isocyanide 1f

EcoScale: 100 – sum of penalty points

Table S2. Calculation of Ecoscale score[a]

Reagents	MF	MW	G	mmol	Equiv.
N-Benzylformamide	C8H9NO	135.16	0.1352	1	1
p-Toluenesulfonyl chloride	C7H7ClO2S	190.64	0.2860	1.5	1.5
Triethylamine	C6H15N	101.19	0.1012	1	1
Sodium carbonate	CNa2O3	105.99	0.6360	6	6
**Product**	**MF**	**MW**	**G**	**mmol**	**Yield**
Benzyl isocyanide	C8H7N	117.15	0.1102	0.94	94%

Entry	Parameters	Penalty Points
1	Yield (94%)	-3
2	Price/availability	-5
3	Safety	-10
4	Technical set-up (Common set-up)	0
5	Temperature/time (r.t.; < 24 h)	-1
6	Work-up and purification	0
	**EcoScale Score**	81

[a]Values calculated using the eco scale calculator software available at the link: http://ecoscale.cheminfo.org/calculator

Calculation of the Green Chemistry metrics for the in-solution preparation of **1f**

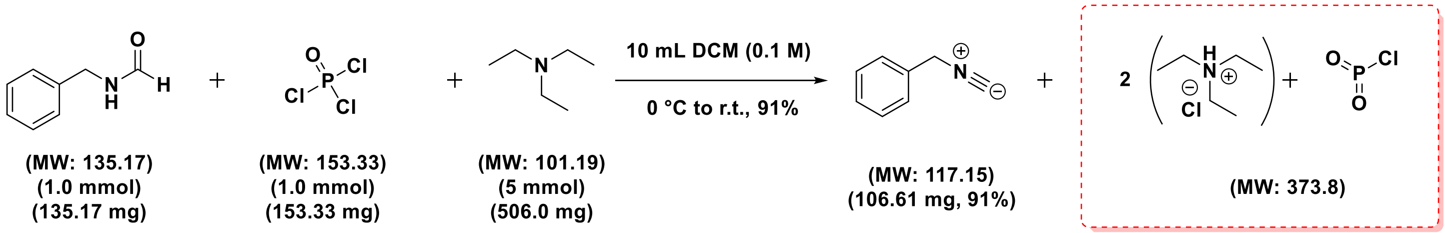

**Excess Reagents**	**-**	**303.57 mg**	**13300 mg (DCM, 10 mL)**

Scheme S2. In-solution* preparation of Isocyanide **1f**.

*Patil, P., Moghaddam, M. A., Dömling, A., *Green Chem.* **2020**. *22*, 6902-6911.

Calculation of green chemistry metrics

$$\textbf{A}\text{tom }\textbf{E}\text{conomy} = \frac{\text{Mass of desired useful product}}{\text{Total Mass of all reactants}} \times 100 = \frac{117{,}15}{389{,}68} \times 100 = 30\%$$

$$\textbf{E}\text{nvironmental }\textbf{F}\text{actor} = \frac{\text{Mass of total waste}}{\text{Mass of desired product}} = \frac{303.6 + 13300{,}0 + 373.8}{106{,}6} = 131.1$$

$$\textbf{R}\text{eaction }\textbf{M}\text{ass }\textbf{E}\text{fficiency} = \frac{\text{actual mass of desired product}}{\text{mass of reactants}} \times 100$$

$$\textbf{RMS} = \frac{106{,}61}{135{,}16+505{,}96+153{,}33} \times 100 = 13{,}42\%$$

The eco-scale score for the in-solution preparation of isocyanide 1f

EcoScale: 100 – sum of penalty points

Table S3. Calculation of Ecoscale score[a]

Reagents	MF	MW	G	mmol	Equiv.
N-Benzylformamide	C_8H_9NO	135.16	0.1352	1	1
Phosphorus oxychloride	Cl_3OP	153.33	0.15333	1	1
Triethylamine	$C_6H_{15}N$	101.19	0.5060	5	5
**Product**	**MF**	**MW**	**G**	**mmol**	**Yield**
Benzyl isocyanide	C_8H_7N	117.15	0.1066	0.91	91%

Entry	Parameters	Penalty Points
1	Yield (91%)	-4
2	Price/availability	-5
3	Safety	-20
4	Technical set-up (Unconventional activation technique)	-2
5	Temperature/time (Cooling to 0 °C)	-4
6	Work-up and purification (Classical chromatography)	-10
	**EcoScale Score**	55

[a]Values calculated using the eco scale calculator software available at the link: http://ecoscale.cheminfo.org/calculator

5. References

[1] Mocci, R.; Murgia, S.; De Luca, L.; Colacino, E.; Delogu, F.; Porcheddu, A. *Org. Chem. Front.* **2018**, *5*, 531–538. doi:10.1039/c7qo01006k

[2] Boeyens, J. C. A., Cook, L. M., Ding, Y., Fernandez, M. A., Reid, D.H., *Org. Biomol. Chem.,* **2003**. *1*, 2168-2172.

[3] https://scifinder-n.cas.org/searchDetail/substance/623ef9727c008e5975793f8b/substanceSpectra.

[4] Kalinski, C., Umkehrer, M., Gonnard, S., Jäger, N., Ross, G., Hiller, W., *Tetrahedron Letters*, **2006**, *47*, 2041-2044.

6. Copies of spectra

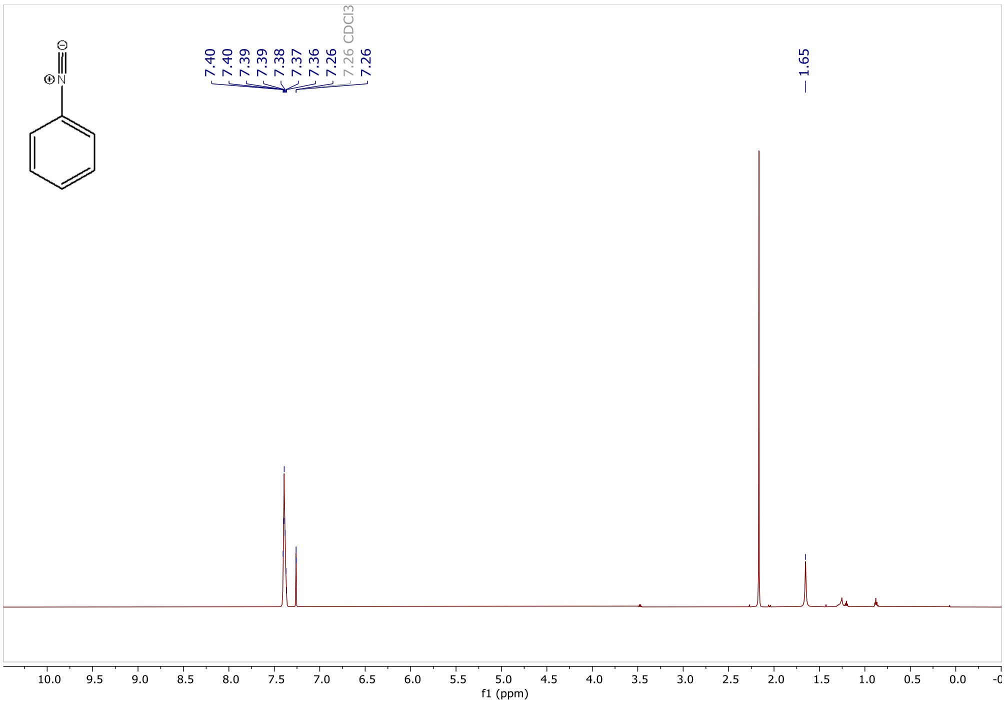

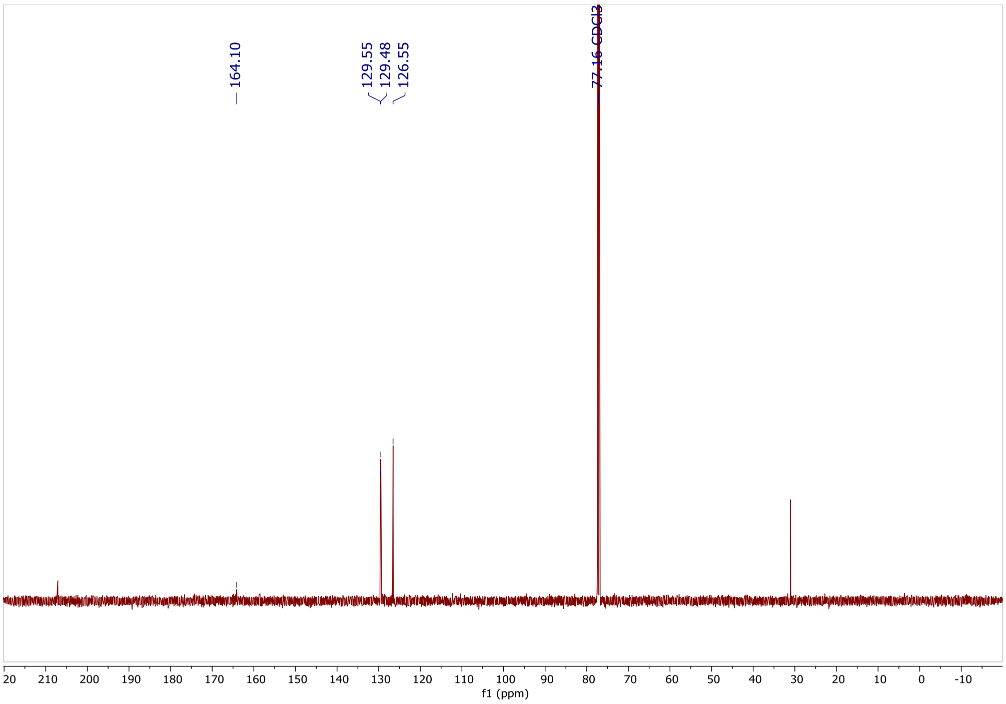

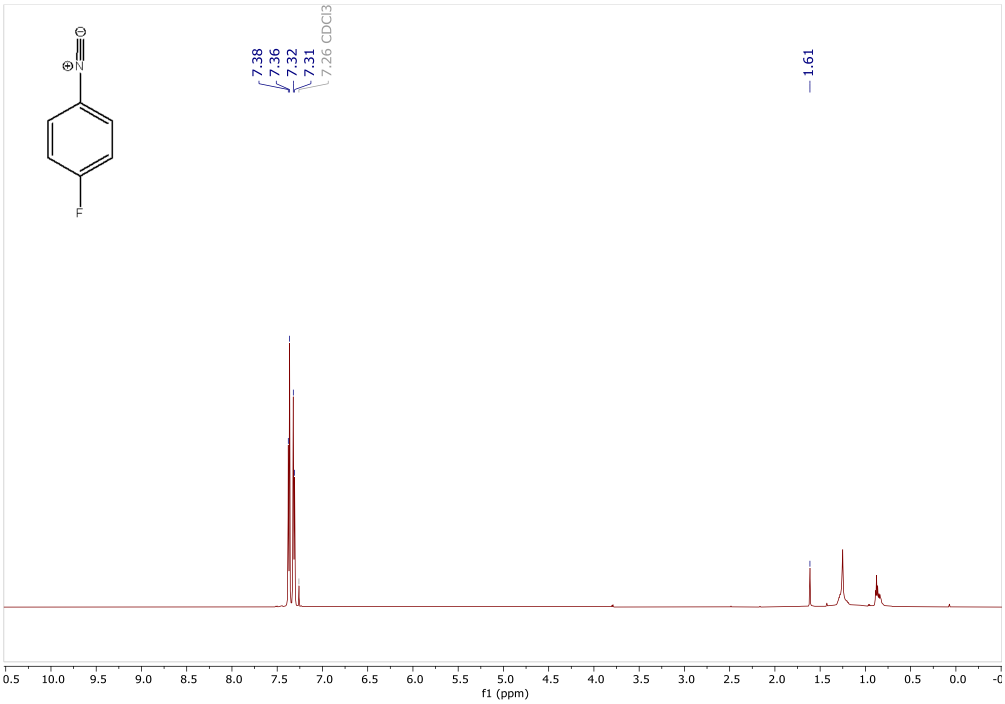
7.38
7.36
7.32
7.31
7.26 CDCl3
1.61
F
f1 (ppm)

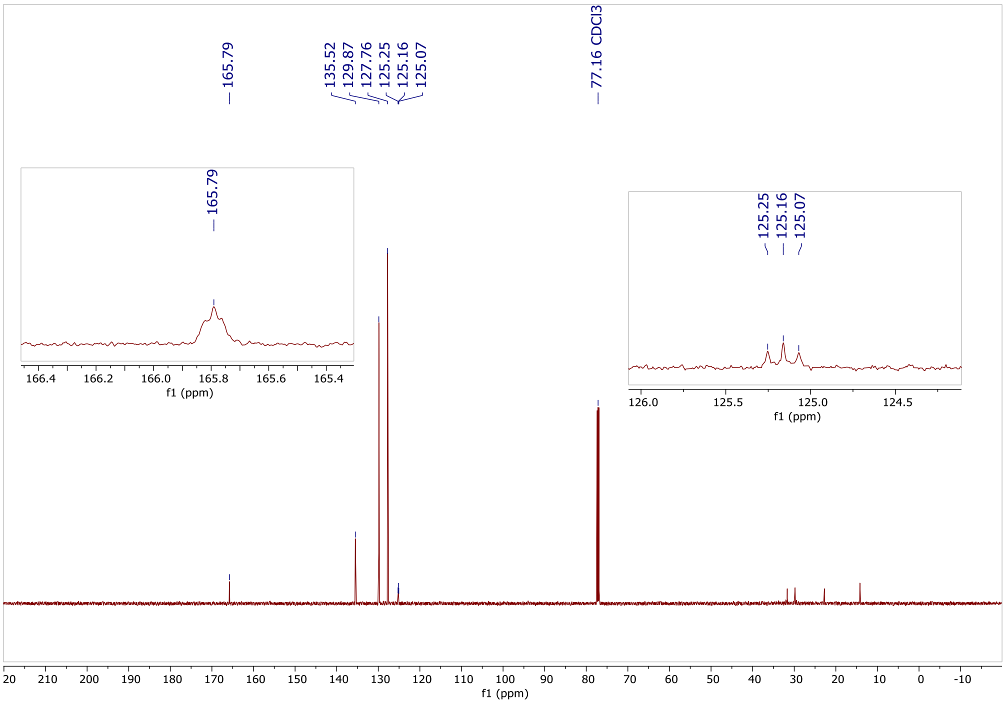
165.79
135.52
129.87
127.76
125.25
125.16
125.07
77.16 CDCl3
f1 (ppm)

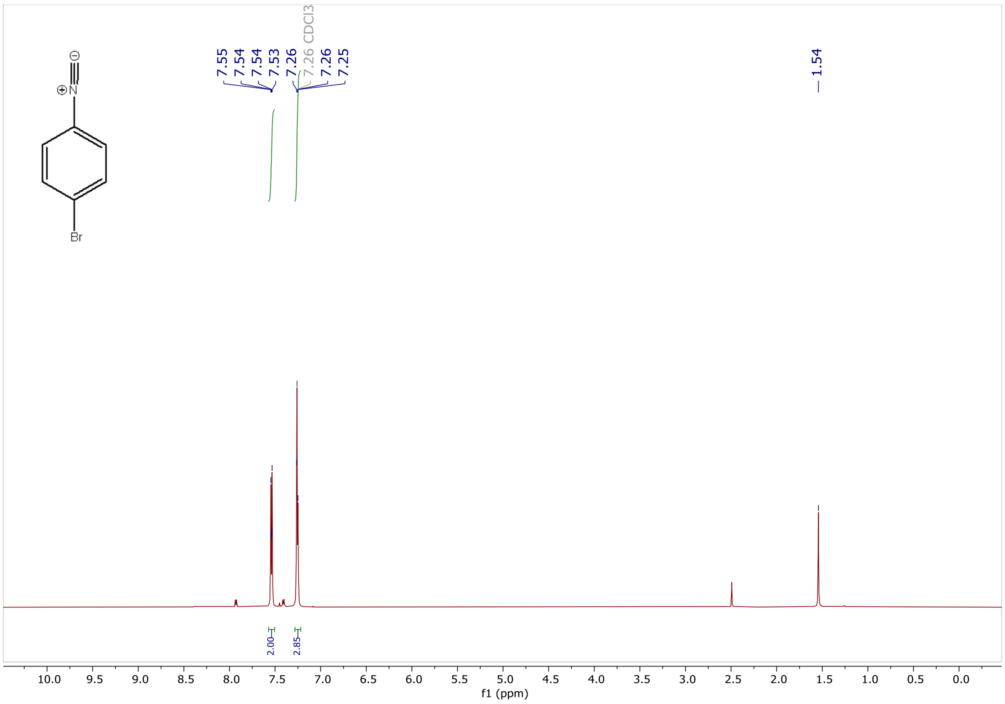
7.55
7.54
7.54
7.53
7.26
7.26 CDCl3
7.26
7.25
1.54
Br
2.00
2.85
10.0 9.5 9.0 8.5 8.0 7.5 7.0 6.5 6.0 5.5 5.0 4.5 4.0 3.5 3.0 2.5 2.0 1.5 1.0 0.5 0.0
f1 (ppm)

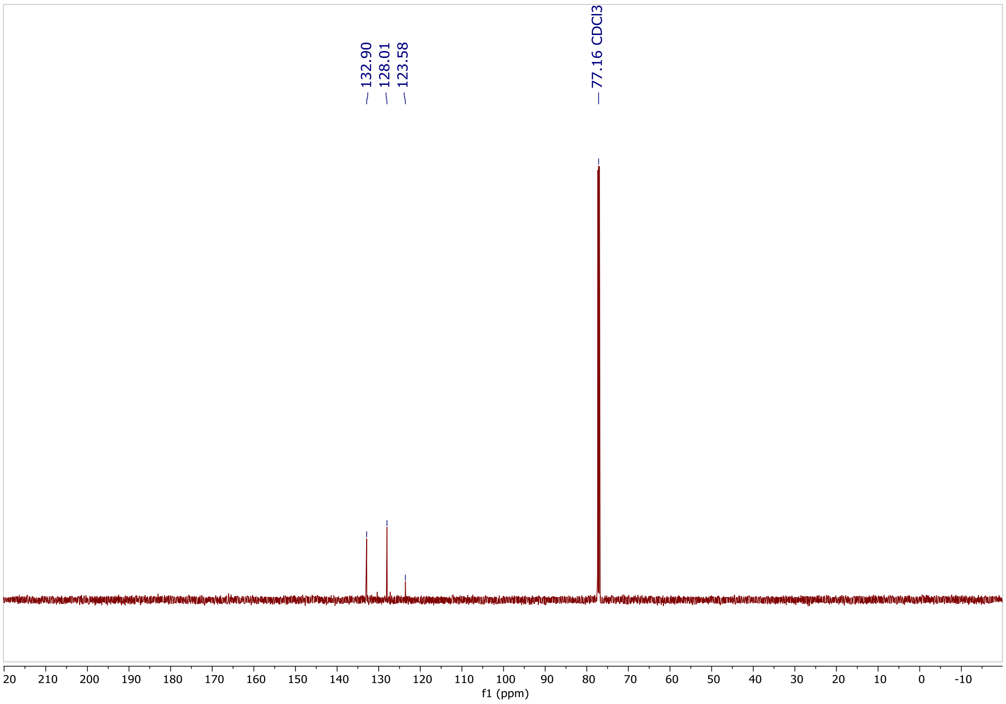
132.90
128.01
123.58
77.16 CDCl3
20 210 200 190 180 170 160 150 140 130 120 110 100 90 80 70 60 50 40 30 20 10 0 -10
f1 (ppm)

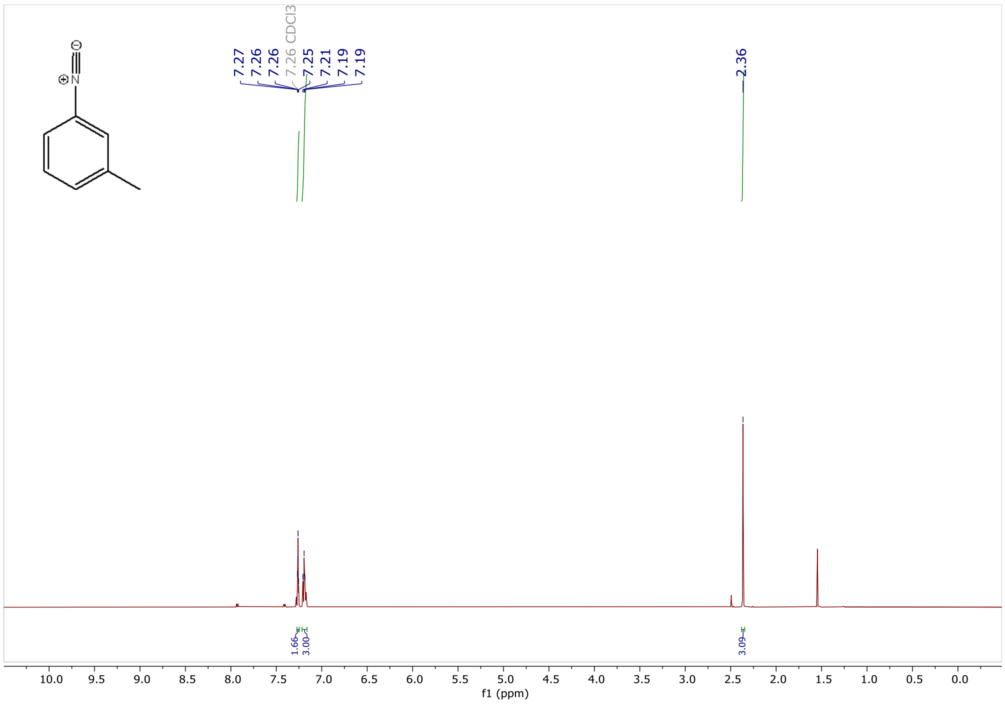
7.27
7.26
7.26 CDCl3
7.25
7.21
7.19
7.19
2.36
1.66
3.00
3.09
10.0 9.5 9.0 8.5 8.0 7.5 7.0 6.5 6.0 5.5 5.0 4.5 4.0 3.5 3.0 2.5 2.0 1.5 1.0 0.5 0.0
f1 (ppm)

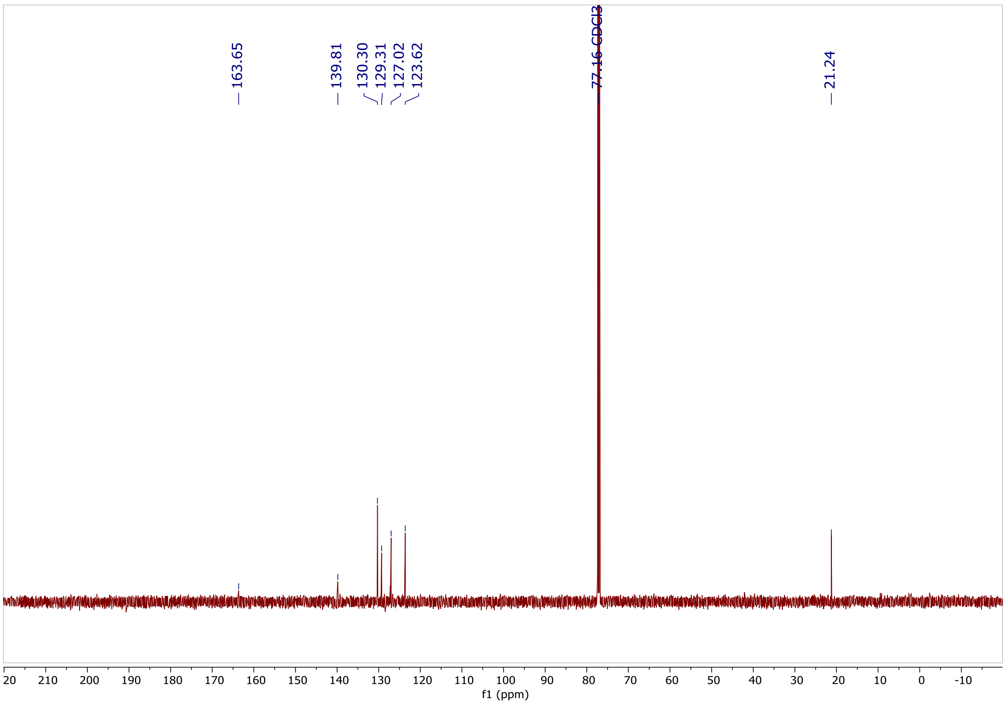
163.65
139.81
130.30
129.31
127.02
123.62
77.16 CDCl3
21.24
210 200 190 180 170 160 150 140 130 120 110 100 90 80 70 60 50 40 30 20 10 0 -10
f1 (ppm)

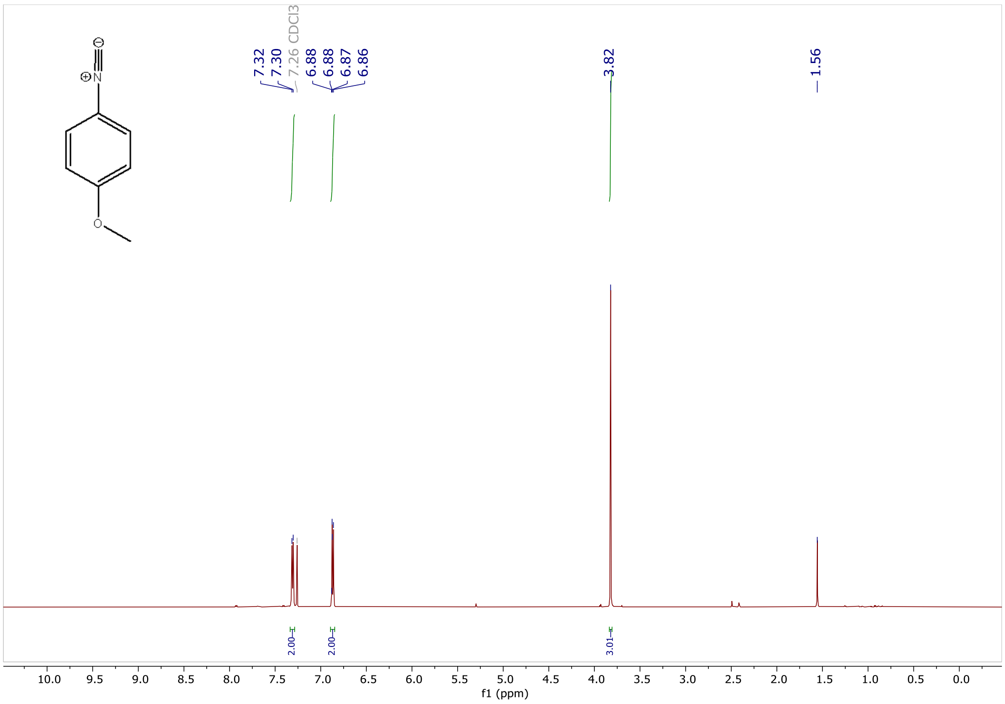
7.32
7.30
7.26 CDCl3
6.88
6.88
6.87
6.86
3.82
1.56
2.00
2.00
3.01
10.0 9.5 9.0 8.5 8.0 7.5 7.0 6.5 6.0 5.5 5.0 4.5 4.0 3.5 3.0 2.5 2.0 1.5 1.0 0.5 0.0
f1 (ppm)

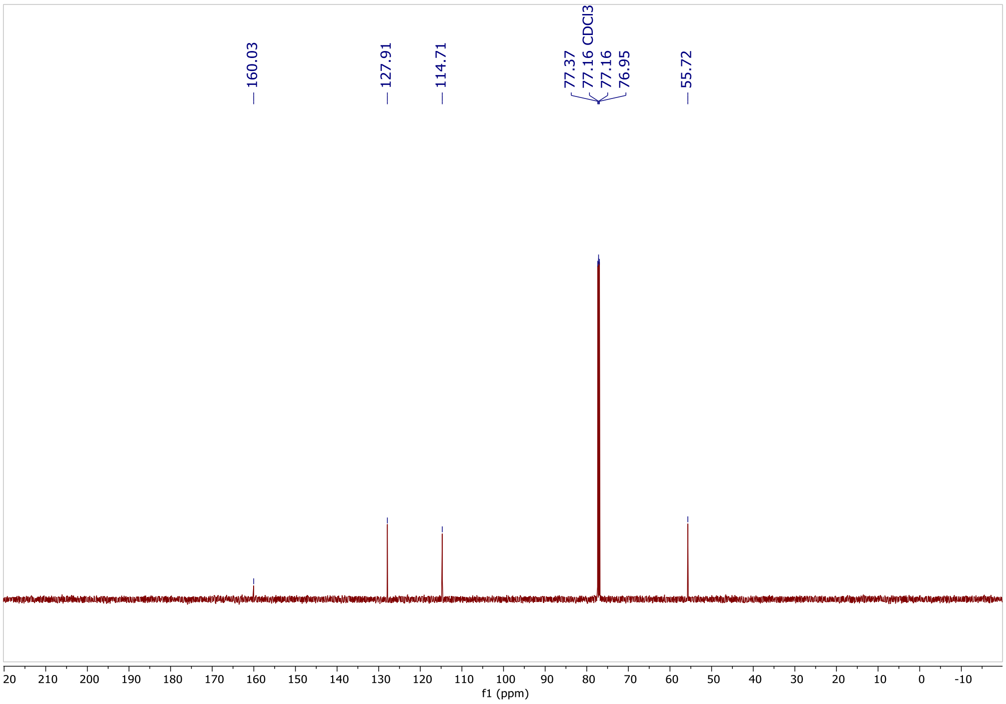
160.03
127.91
114.71
77.37
77.16 CDCl3
77.16
76.95
55.72
20 210 200 190 180 170 160 150 140 130 120 110 100 90 80 70 60 50 40 30 20 10 0 -10
f1 (ppm)

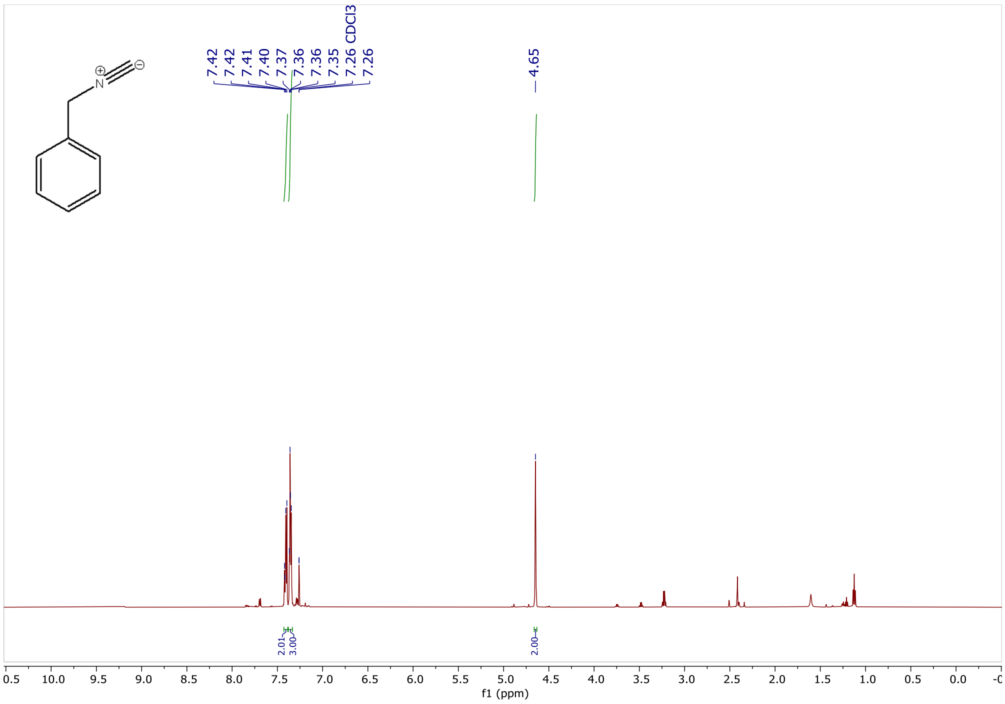
7.42
7.42
7.41
7.40
7.37
7.36
7.36
7.35
7.26 CDCl3
7.26
4.65
2.01
3.00
2.00
f1 (ppm)

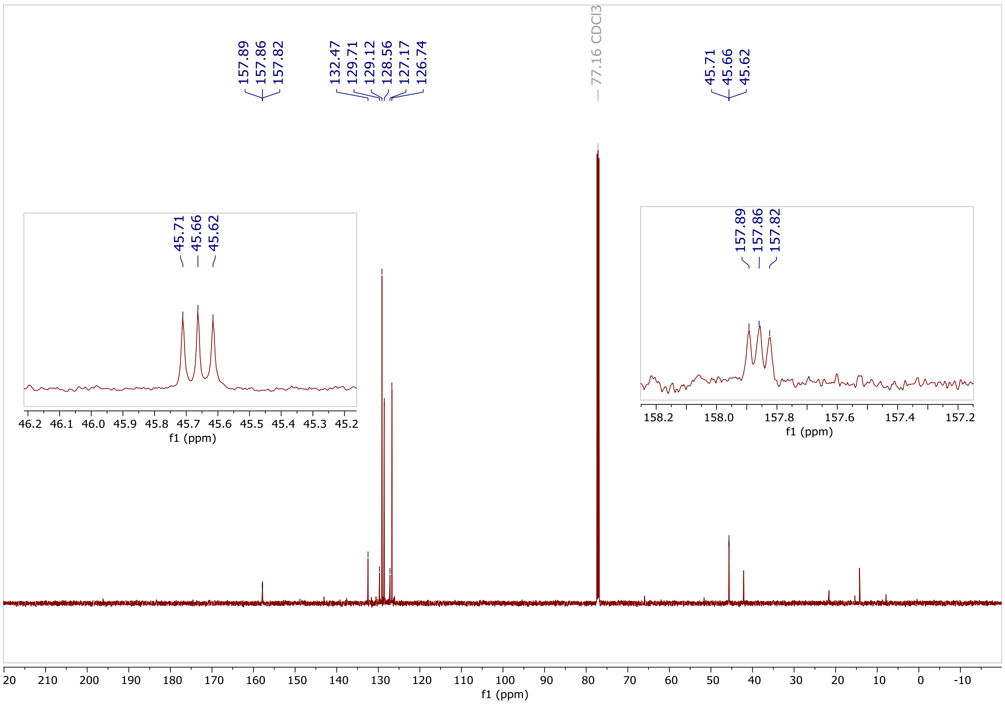
157.89
157.86
157.82
132.47
129.71
129.12
128.56
127.17
126.74
77.16 CDCl3
45.71
45.66
45.62
f1 (ppm)

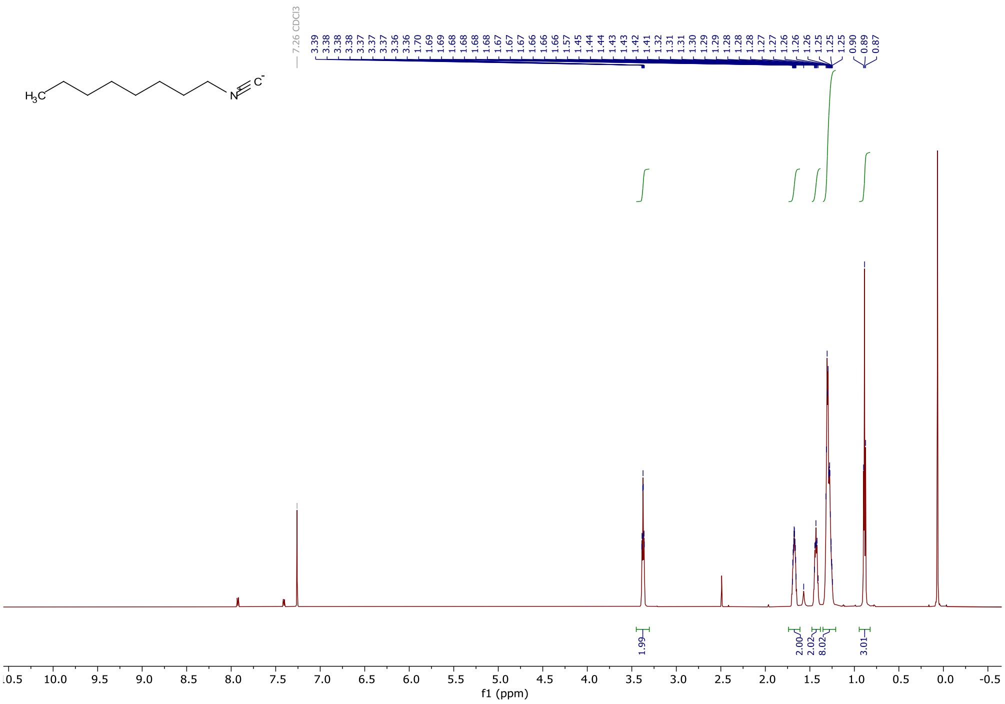

7.26 CDCl3
1.99
2.00
2.02
8.02
3.01
f1 (ppm)

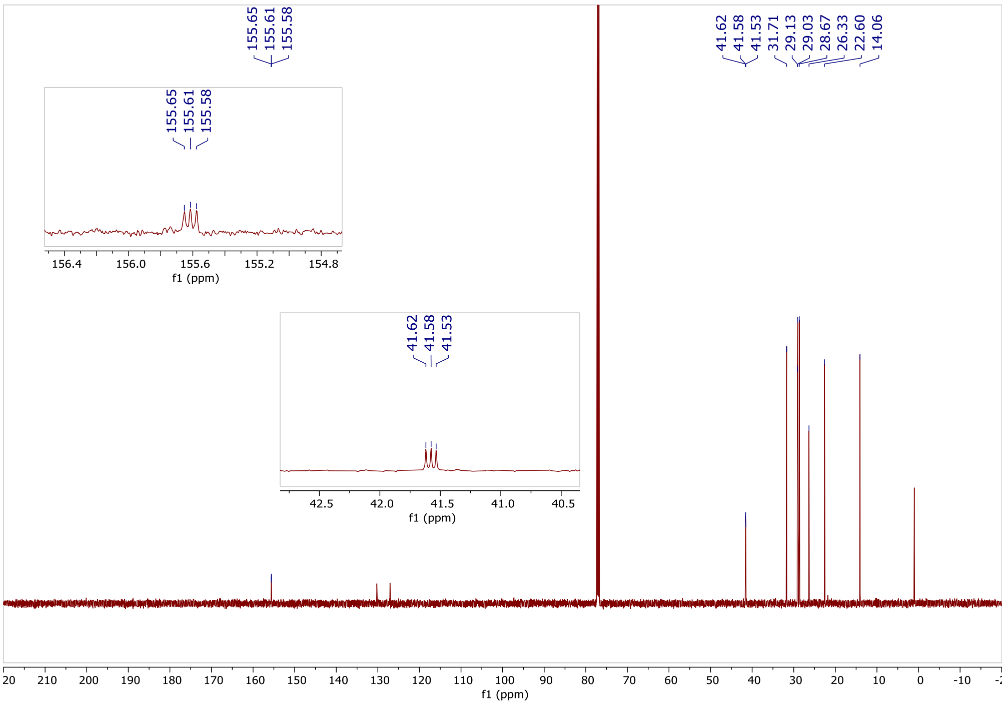

155.65
155.61
155.58
41.62
41.58
41.53
31.71
29.13
29.03
28.67
26.33
22.60
14.06
f1 (ppm)

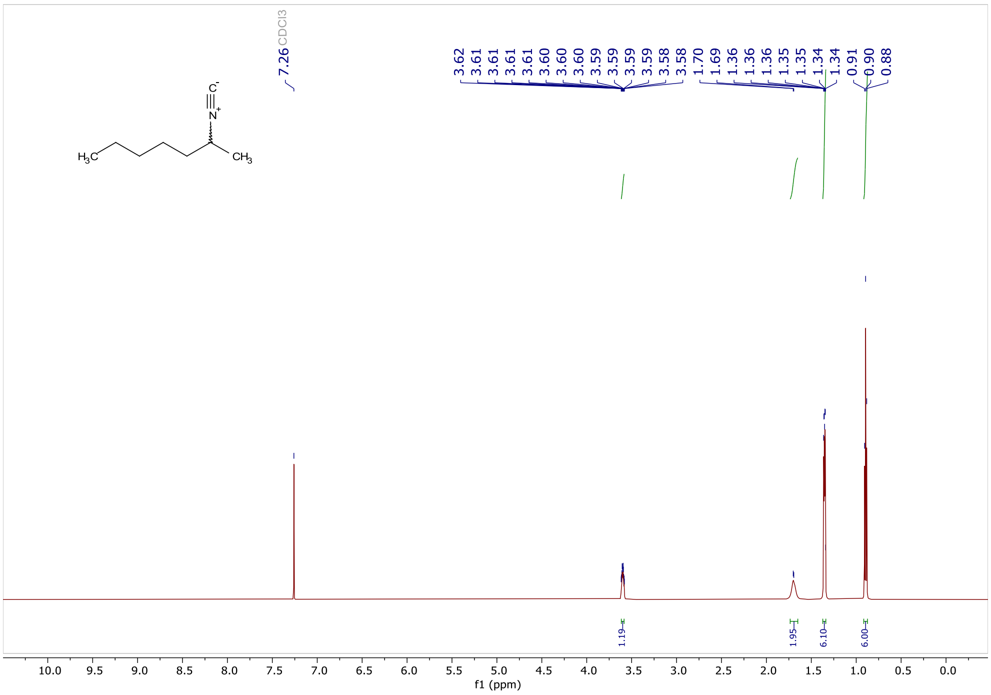

7.26 CDCl3
3.62
3.61
3.61
3.61
3.61
3.60
3.60
3.59
3.59
3.59
3.59
3.58
3.58
1.70
1.69
1.36
1.36
1.36
1.35
1.35
1.34
1.34
0.91
0.90
0.88
1.19
1.95
6.10
6.00
f1 (ppm)

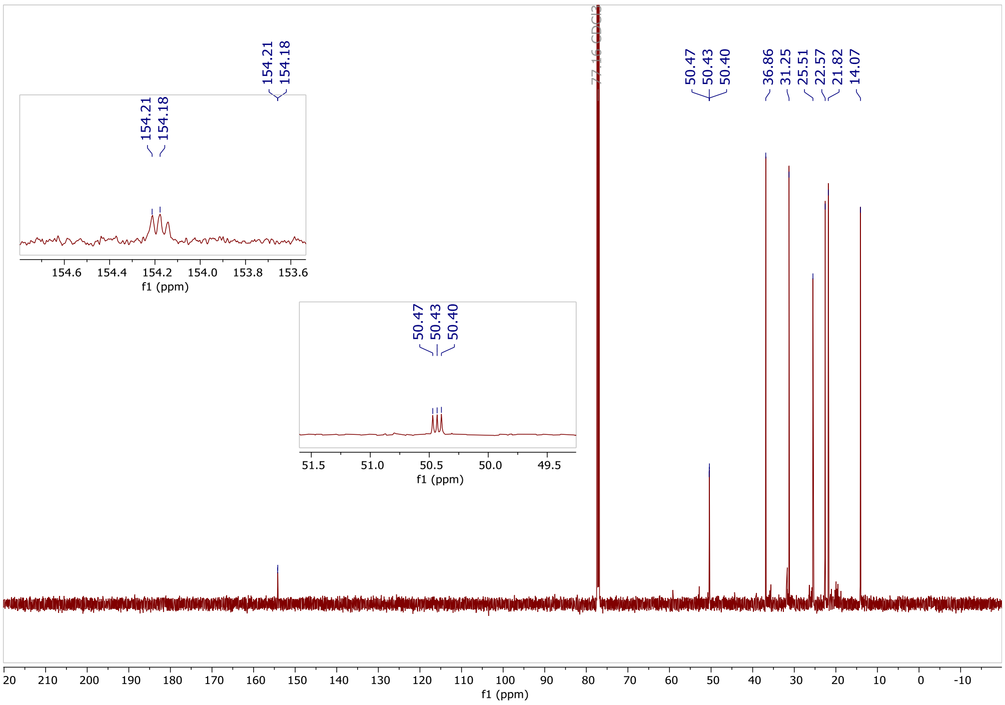

154.21
154.18
50.47
50.43
50.40
36.86
31.25
25.51
22.57
21.82
14.07
f1 (ppm)

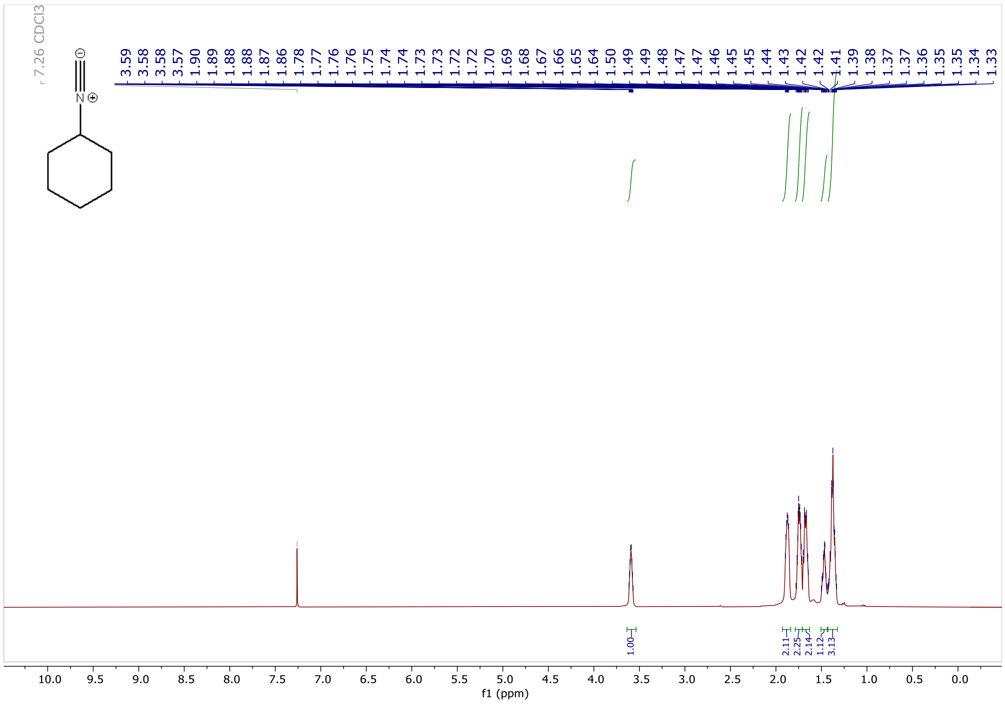

7.26 CDCl3
3.59
3.58
3.58
3.57
1.90
1.89
1.88
1.88
1.87
1.86
1.78
1.77
1.76
1.76
1.75
1.74
1.74
1.73
1.73
1.72
1.72
1.70
1.69
1.68
1.67
1.66
1.65
1.64
1.50
1.49
1.49
1.48
1.47
1.47
1.46
1.45
1.45
1.44
1.43
1.42
1.42
1.41
1.39
1.38
1.37
1.37
1.36
1.35
1.35
1.34
1.33
N
1.00
2.11
2.25
2.14
1.12
3.13
f1 (ppm)

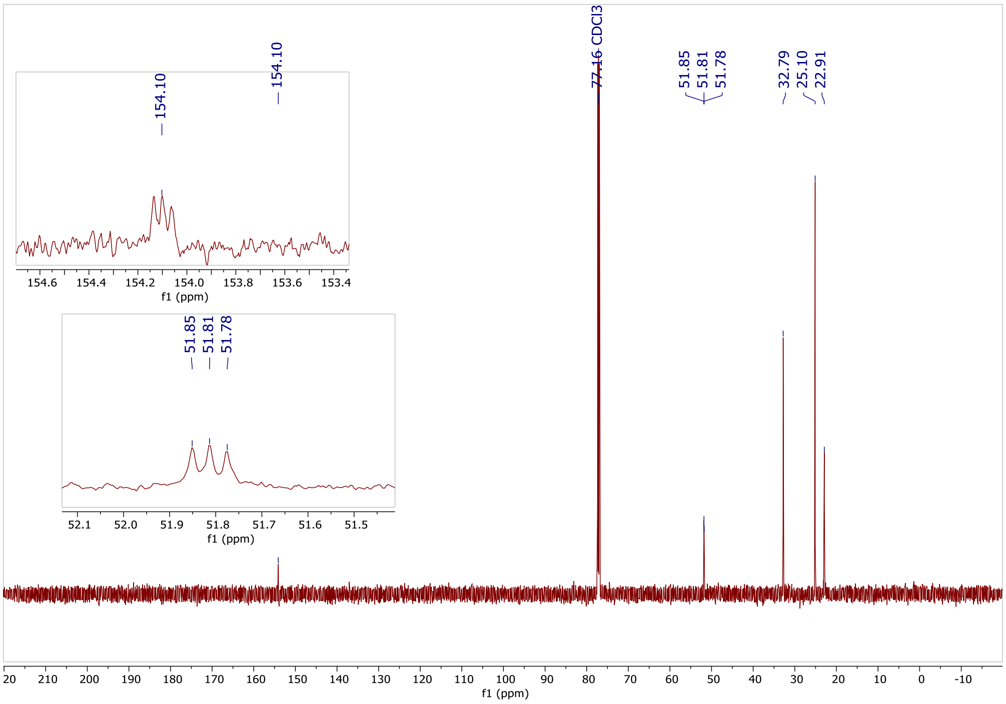

77.16 CDCl3
51.85
51.81
51.78
32.79
25.10
22.91
154.10
f1 (ppm)
